# Disruption of Tumor Necrosis Factor Receptor-Associated Factor 5 Exacerbates Murine Experimental Colitis via Regulating T Helper Cell-Mediated Inflammation

**DOI:** 10.1155/2016/9453745

**Published:** 2016-03-24

**Authors:** Jian Shang, Lixia Li, Xiaobing Wang, Huaqin Pan, Shi Liu, Ruohang He, Jin Li, Qiu Zhao

**Affiliations:** ^1^Department of Gastroenterology/Hepatology, Zhongnan Hospital of Wuhan University, Wuhan 430071, China; ^2^The Hubei Clinical Center & Key Laboratory of Intestinal & Colorectal Diseases, Wuhan 430071, China

## Abstract

Tumor necrosis factor (TNF) receptor-associated factor 5 (TRAF5) is a key mediator of TNF receptor superfamily members and is important in both T helper (Th) cell immunity and the regulation of multiple signaling pathways. To clarify TRAF5's influence on inflammatory bowel diseases (IBDs), we investigated TRAF5 deficiency's effect on dextran sulfate sodium- (DSS-) induced colitis. Colitis was induced in TRAF5 knockout (KO) mice and their wild-type (WT) littermates by administering 3% DSS orally for 7 days. The mice were then sacrificed, and their colons were removed. Our data suggested that KO mice were more susceptible to DSS-induced colitis. TRAF5 deficiency significantly enhanced IFN-*γ*, IL-4, and IL-17a mRNA and protein levels in the colons of DSS-fed mice, and the mRNA expression of T-bet and GATA-3 was also markedly elevated. However, ROR-*α* and ROR-*γ*t mRNA levels did not differ between DSS-induced KO and WT mice. Flow cytometry showed increased frequencies of Th2 and IFN-*γ*/IL-17a-coproducing CD4^+^ T cells in the colons of DSS-induced KO mice. Additionally, TRAF5 deficiency significantly enhanced the activation of NF-*κ*B in CD4^+^ T cells after DSS administration. These results indicated that TRAF5 deficiency significantly aggravated DSS-induced colitis, most likely by regulating Th cell-mediated inflammation.

## 1. Introduction

Inflammatory bowel diseases (IBDs), which consist of Crohn's disease (CD) and ulcerative colitis (UC), are chronic and complicated inflammatory disorders of the gastrointestinal tract [1]. Although the exact etiology of IBDs remains elusive, numerous studies have suggested that CD4^+^ T helper (Th) cells play important roles in both the induction and the maintenance of IBDs [2, 3]. Th cells are traditionally divided into three subsets: Th1, Th2, and Th17. UC is generally considered to be a Th2-type disease, as indicated by elevated levels of interleukin-4 (IL-4), IL-5, and IL-13. In contrast, in CD, it has been suggested that Th1 and Th17 cells play essential roles in the disease's development [4–6].

Feeding mice with dextran sulfate sodium (DSS) solution continuously for several days can induce an acute colitis that is characterized by diarrhea, rectal bleeding, body weight loss, shortening of the colon, and mucosal ulceration. This relatively simple and replicable method by which chemical colitis in mice is induced has been widely used as an experimental animal model of human IBDs and especially UC [7, 8].

Tumor necrosis factor (TNF) receptor-associated factors (TRAFs) represent a group of important signaling transducers for the IL-1, TNF, and Toll-like receptor (TLR) superfamilies. Structurally, TRAFs consist of an N-terminal cysteine/histidine-rich region as well as a homologous area at the C-terminus [9]. Numerous studies have suggested that TRAFs participate in multiple biological functions, such as tumorigenesis, stress reactions, innate immunity, and adaptive immunity [10–13]. To date, seven members, named TRAF1 to TRAF7, have been identified in the TRAF family, although TRAF5 is relatively less understood [9, 14]. Although TRAF5 shares obvious structural homology with TRAF3, its molecular function is most similar to that of TRAF2 [13, 15, 16]. In contrast to mice lacking TRAF2 or TRAF3, which die prematurely, TRAF5 knockout (KO) mice grow vigorously and display no obvious sign of abnormality [17]. TRAF5 has been suggested to be an important regulator of Th cell immunity and also plays essential roles in mediation of the nuclear factor-kappa B (NF-*κ*B) signaling pathway [15, 18–22]. However, the exact contribution of TRAF5 to IBDs is still unclear.

In the present study, for the first time, we used 3% DSS to induce acute colitis in TRAF5 KO mice and their wild-type (WT) littermates to determine the specific roles of TRAF5 in the pathogenesis of IBDs.

## 2. Materials and Methods

### 2.1. Ethics Statement

In our study, all of the animal experiments were conducted according to the recommendations in the Guide for the Care and Use of Laboratory Animals of Wuhan University, and the protocols were approved by the Committee on the Ethics of Animal Experiments of Wuhan University.

### 2.2. Animals

Breeding pairs of TRAF5 KO mice (C57BL/6 background) were a generous gift from Professor Hongliang Li (Cardiovascular Research Institute, Wuhan University). TRAF5 KO mice were backcrossed with C57BL/6 mice for 6 or more generations, and the heterozygous parental mice were intercrossed to yield TRAF5^−/−^ and TRAF5^+/+^ offspring that were used for further study. The animals in our experiments were bred and maintained under specific pathogen-free (SPF) conditions and had ad libitum access to food and water. The mice were acclimated to these conditions for more than 5 days before the start of the experiments. All of the mice used were male, aged 8–10 weeks, and had a body weight of 20–24 g.

### 2.3. Induction and Evaluation of Colitis

Acute colitis was induced in TRAF5^−/−^ and TRAF5^+/+^ mice (*N* = 10 in each group) using 3% DSS (MW 36,000–50,000, MP Biomedicals, Solon, OH) dissolved in drinking water for 7 days. Normal control mice were given distilled water throughout the experiments. The animals were observed once daily for body weight, stool consistency, and the presence of occult or gross blood in the feces. The disease activity index (DAI) was calculated as described by Cooper et al. (Table 1) [23]. At the end of the experiments, the mice were sacrificed by CO_2_ inhalation. Postmortem, the entire colons were rapidly removed, photographed, and gently cleared of feces using 4°C normal saline. Small segments of the distal colons collected for histopathology and immunofluorescence analysis were fixed in 10% normal buffered formalin. The remaining colon tissues were stored at −80°C for use in the remaining experiments.

### 2.4. Histological Analysis

Colon segments for histopathology were fixed in 10% formalin, as mentioned above. After paraffin embedding, 5 *μ*m sections were cut and stained with hematoxylin and eosin (H&E) in accordance with standard protocols. Histological scoring was conducted blindly by two pathologists. According to Obermeier et al. [24], the scoring was based on a combination of epithelial damage and inflammatory cell infiltration (Table 2).

### 2.5. Myeloperoxidase (MPO) Activity Assay

MPO is an enzyme found mainly in granulocytes, and its activity was assessed using an MPO assay kit (Jiancheng Bioengineering, Nanjing, China) according to the manufacturer's instructions. Briefly, the MPO activities of the colon samples were detected by utilizing a kinetic assay in which H_2_O_2_ was degraded by the MPO released from neutrophil granulocytes. The absorbance at 460 nm was then measured using a DU 530 Life Science UV/Vis Spectrophotometer (Beckman Coulter, USA). The activity of the MPO is shown in units per gram of tissue (U/g tissue).

### 2.6. RNA Extraction and Quantitative Real-Time Polymerase Chain Reaction (qRT-PCR)

Total RNA was extracted from the colon samples using TRIzol reagent (Invitrogen, USA) according to the manufacturer's protocols. RNA quantification was performed using a spectrophotometer (NanoDrop 2000, Thermo Scientific, USA). cDNA was then synthesized using a first-strand cDNA synthesis kit (Thermo Scientific, USA) and 2 *μ*g total RNA. qRT-PCR was subsequently conducted using the QuantStudio*™* 6 Flex Real-Time PCR System (ABI, USA) and SYBR® Premix Ex Taq*™* II mix (Takara, Japan). The gene-specific primer pairs used are listed in Table 3. The 2^−ΔΔCT^ method was used to determine the relative mRNA levels.

### 2.7. Measurement of Colonic Cytokines

Colon tissues taken from KO and WT mice were weighed and homogenized in phosphate-buffered saline (PBS) containing 0.05% Tween-20, 0.1 mM phenylmethylsulfonyl fluoride (PMSF), 0.1 mM benzethonium chloride, 10 mM EDTA, and 20 KIU aprotinin. Subsequently, the tissue homogenates were centrifuged at 12,000 g for 10 min, and the supernatants were removed and stored at −80°C until use. Quantification of the cytokines IFN-*γ*, IL-4, and IL-17a was then performed using commercially available enzyme-linked immunosorbent assay (ELISA) kits (Joyee Biotechnics, Shanghai, China) according to the manufacturer's protocols.

### 2.8. Immunofluorescence Staining

For immunofluorescence staining, 5 *μ*m sections cut from paraffin-embedded tissues were deparaffinized, incubated with sodium citrate buffer (10 mM, pH 6.0) for antigen retrieval, and then blocked with 5% goat serum for 30 min at room temperature. Subsequently, the slides were incubated with specific primary antibodies overnight at 4°C. The following primary antibodies were used: anti-IFN-*γ* (1 : 200 dilution; Proteintech, USA), anti-IL-4 (1 : 100 dilution; BioLegend, USA), anti-IL-17a (1 : 200 dilution; Proteintech, USA), anti-p65 (1 : 100 dilution; Cell Signaling Technology, USA), anti-CD4 (1 : 100 dilution; BioLegend, USA), and anti-CK18 (1 : 100 dilution; Goodbio Technology Co., China). After careful washing with PBS, the slides were incubated with appropriate species-specific secondary antibodies for 1 h. Finally, the sections were stained with 4′,6-diamidino-2-phenylindole (DAPI) to visualize the cell nuclei, and visualization was performed using fluorescence microscopy.

### 2.9. Isolation of Lamina Propria Mononuclear Cells (LPMCs)

LPMC suspensions were collected as described, with minor modification [25]. Briefly, the colons were opened longitudinally, cautiously washed with ice-cold PBS, and cut into small pieces (5 mm). To remove epithelial cells and intraepithelial lymphocytes, the colon segments were preincubated in HBSS^−/−^ buffer (without Ca^2+^/Mg^2+^) containing 2 mM EDTA, 10 mM HEPES, 1 mM DTT, and 5% FCS twice for 15 min at 37°C. The remaining pieces were then incubated in HBSS^+/+^ buffer (with Ca^2+^/Mg^2+^) containing 1.5 mg/mL collagenase VIII (Sigma-Aldrich, USA), 0.1 mg/mL DNase I (Thermo Scientific, USA), and 5% FCS for 45 min at 37°C. After filtration of the digested tissue through a mesh filter, the isolated cells were purified using 40% and 80% Percoll gradients (Sigma-Aldrich, USA) by centrifugation at 1,000 g for 20 min. The LPMCs were then collected at the interface of the 40% and 80% Percoll gradients.

### 2.10. Intracellular Staining

For staining for intracellular IFN-*γ*, IL-4, and IL-17a, the obtained LPMCs were stimulated with Leukocyte Activation Cocktail (BD Biosciences, USA) for at least 5 h in vitro. The cells were then harvested and stained with phycoerythrin-cyanine 7- (PE-Cy7-) conjugated anti-CD4 antibody (eBioscience, USA) for 30 min. Subsequently, the cells were fixed and permeabilized using the BD Cytofix/Cytoperm*™* Fixation/Permeabilization Kit (BD Biosciences, USA) according to the manufacturer's instructions, followed by staining with allophycocyanin- (APC-) conjugated anti-IFN-*γ* antibody, phycoerythrin- (PE-) conjugated anti-IL-4 antibody, and PE-conjugated anti-IL-17a antibody (all purchased from BD Biosciences, USA). Finally, the cells were analyzed using a FACSCalibur flow cytometer (BD Biosciences, USA). The threshold of positivity was defined beyond the nonspecific binding observed in the presence of a relevant isotype-control antibody.

### 2.11. SDS-Polyacrylamide Gel Electrophoresis (PAGE) and Western Blotting

Colon tissues taken from KO and WT mice were lysed in RIPA lysis buffer containing protease inhibitor cocktail, and the protein concentration was detected using a BCA protein assay kit (Beyotime Biotechnology, China). A total of 50 *μ*g protein was then loaded and separated on a 12% SDS-PAGE gel and transferred to polyvinylidene difluoride (PVDF) membranes (Millipore) using a wet-transfer apparatus. The membranes were blocked in TBST (Tris-buffered saline with Tween-20) containing 5% nonfat milk powder for 1 h at room temperature, followed by incubation with specific primary antibodies overnight at 4°C. After 3 washes in TBST, the membranes were incubated with horseradish peroxidase- (HRP-) conjugated species-specific antibodies for 2 h at room temperature. After another 3 washes with TBST, the blots were visualized using high-sensitivity chemiluminescence, followed by exposure to autoradiography film. Density analyses of the blots were performed using Quantity One software (Bio-Rad). Specific protein expression levels on the same membrane were normalized to GAPDH levels. The following primary antibodies were used: anti-phospho-p65 (no. 3033), anti-total-p65 (no. 8242), anti-phospho-I*κ*B*α* (no. 2859), and anti-total-I*κ*B*α* (no. 4814) (1 : 1000 dilution; all from Cell Signaling Technology, Beverly, MA, USA). The antibody against GAPDH was purchased from Sungene Biotech (Tianjin, China).

### 2.12. Statistical Analysis

All data are presented as the mean ± SD. Statistically significant differences were analyzed using one-way analysis of variance (ANOVA) or an unpaired* t*-test. A *p* value less than 0.05 was considered significant. All statistical analyses were conducted using SPSS 17.0 software.

## 3. Results

### 3.1. TRAF5-Deficient Mice Develop More Severe Colitis in Comparison with WT Mice in the DSS-Induced Colitis Model

To evaluate the roles of TRAF5 in the development of murine experimental colitis, acute colitis was induced in TRAF5 KO mice and their WT littermates by administering 3% DSS in their drinking water for 7 days.

**Figure 1:**
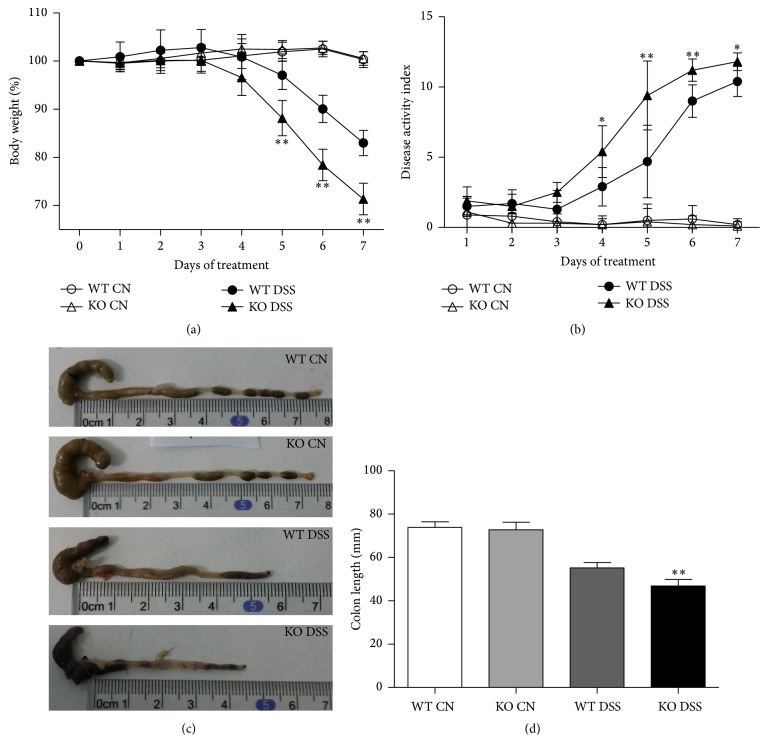
TRAF5-deficient mice are more susceptible to DSS induction. (a) Change in percent body weight. (b) Change in DAI scores. ((c) and (d)) Mice were sacrificed on day 7, and their colons were removed, photographed, and measured in terms of length. The data are presented as the mean ± SD. *N* = 10 per group. ^*∗*^ *p* < 0.05 versus the DSS-treated WT group and ^*∗∗*^ *p* < 0.01 versus the DSS-treated WT group.

During the administration of DSS, TRAF5 KO mice suffered from significantly greater body weight loss beginning on day 5 in comparison with WT mice (day 5: 88.13 ± 3.64% versus 97.04 ± 2.97%, *p* < 0.01; day 6: 78.41 ± 3.25% versus 90.04 ± 2.83%, *p* < 0.01; day 7: 71.32 ± 3.28% versus 82.98 ± 2.63%, *p* < 0.01) (Figure 1(a)). In addition, the DAI scores of TRAF5-deficient mice significantly worsened compared with those of DSS-fed WT mice (day 4, *p* = 0.019; day 5, *p* = 0.003; day 6, *p* = 0.001; day 7, *p* = 0.018) (Figure 1(b)). It is known that colon length is one of the parameters with less flexibility in the DSS-induced colitis model; we found that the colons of KO mice were much shorter than those of WT mice following treatment with DSS (46.9 ± 2.9 mm versus 55.2 ± 2.5 mm, *p* < 0.01) (Figures 1(c) and 1(d)).

**Figure 2:**
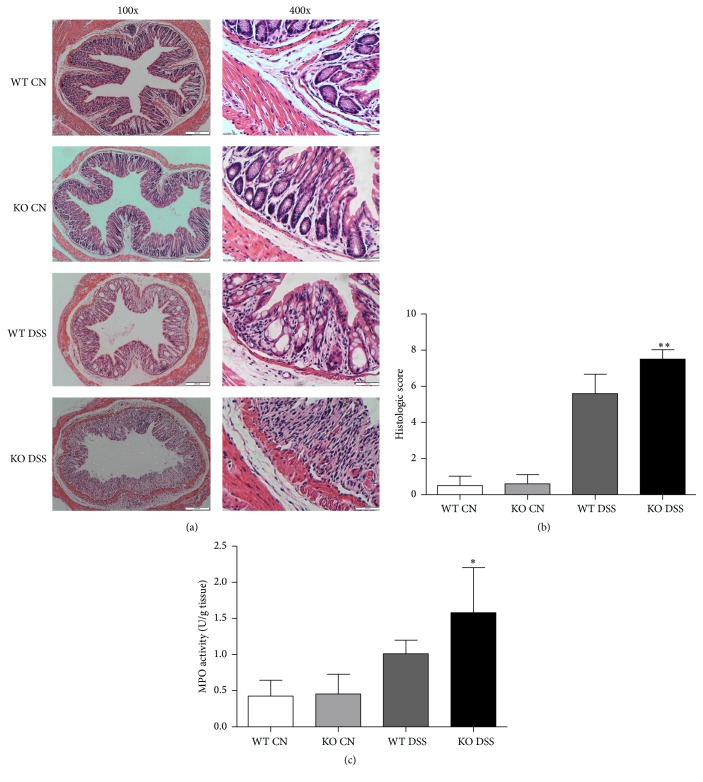
Effect of TRAF5 deficiency on the histopathology and MPO activities of colon tissues. Histological evaluation of DSS-induced colitis was performed by microscopy and H&E staining. Colon sections from TRAF5 KO and WT mice were scored in a blinded fashion, as described in Section 2. (a) Representative cross sections of the distal colon. The magnifications of the images are 100-fold and 400-fold. (b) Quantitative results of the histological analysis (*N* = 10 per group). (c) MPO activities in the colons were determined as described in Section 2 (*N* = 6 per group). The data are presented as the mean ± SD. ^*∗*^ *p* < 0.05 versus the DSS-treated WT group and ^*∗∗*^ *p* < 0.01 versus the DSS-treated WT group.

DSS administration led to loss of the normal colonic architecture, which was characterized by epithelial damage and infiltration of inflammatory cells. Using a comprehensive scoring system to quantify the degree of damage, we observed much more severe histological injury in TRAF5 KO mice compared with WT mice following DSS induction (Figures 2(a) and 2(b)). We also scored the histology of all of the control mice and observed no obvious inflammation in untreated KO or WT mice. Moreover, elevated levels of MPO activities were found in the colons of TRAF5-deficient animals after DSS administration (Figure 2(c)), indicating that the loss of TRAF5 could markedly promote colonic neutrophil infiltration in response to DSS. Our data therefore demonstrated that TRAF5 deficiency could significantly exacerbate the severity of DSS-induced colitis.

### 3.2. TRAF5 Deficiency Alters Cytokine and Transcription Factor Production in the Colons of DSS-Induced Mice

To understand the mechanism that underlies the exacerbation of DSS-induced colitis in TRAF5-deficient mice, we examined the mRNA levels of typical cytokines and transcription factors associated with Th1 (TNF-*α*, IFN-*γ*, and T-bet), Th2 (IL-4 and GATA-3), and Th17 (IL-17a, IL-22, ROR-*α*, and ROR-*γ*t) cells by qRT-PCR.

**Figure 3:**
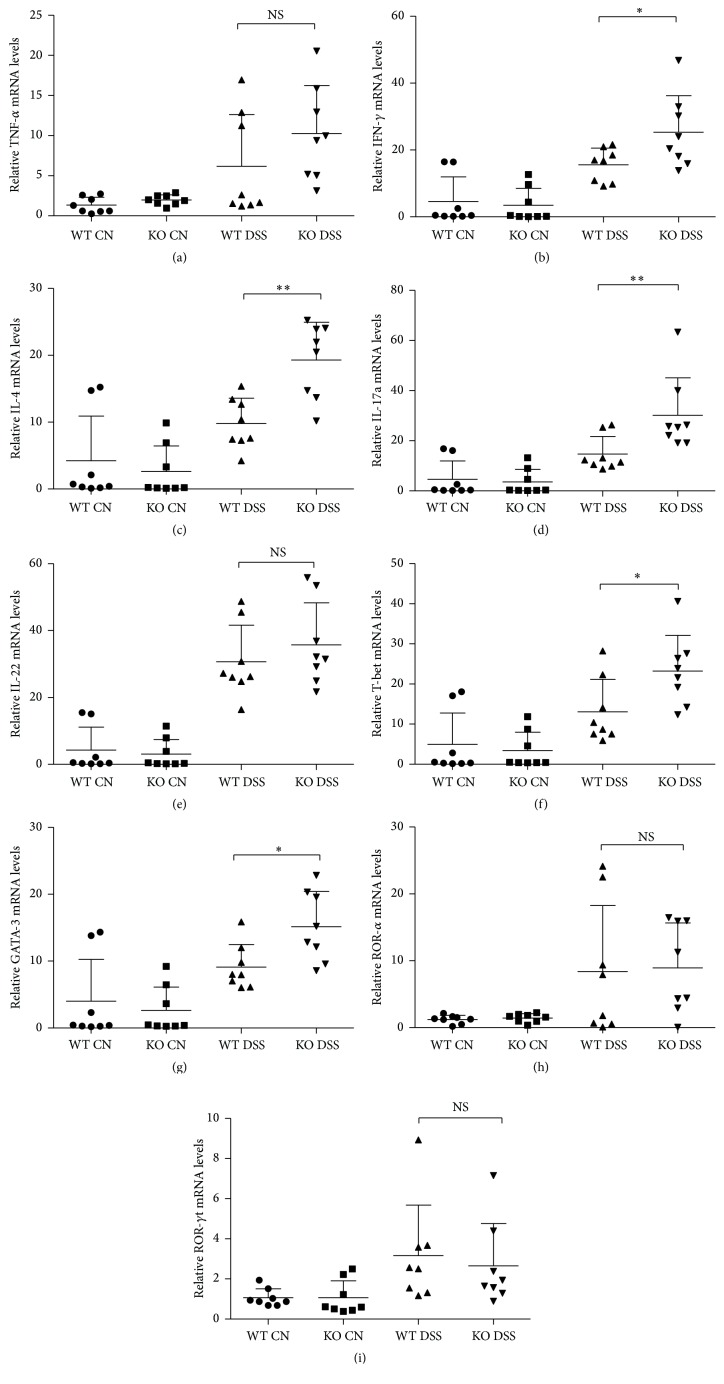
Analysis of the mRNA expression of cytokines and transcription factors for Th cells in the colons of TRAF5 KO and WT mice. (a) TNF-*α*, (b) IFN-*γ*, (c) IL-4, (d) IL-17a, (e) IL-22, (f) T-bet, (g) GATA-3, (h) ROR-*α*, and (i) ROR-*γ*t mRNA expression levels were determined by real-time PCR. The data are representative of 3 independent experiments (mean ± SD). *N* = 8 per group. ^*∗*^ *p* < 0.05 versus the DSS-treated WT group; ^*∗∗*^ *p* < 0.01 versus the DSS-treated WT group. NS: not significant versus the DSS-treated WT group.

Compared with levels in DSS-treated WT mice, the mRNA levels of typical Th1/Th2 cytokines (IFN-*γ* and IL-4, resp.) and transcription factors (T-bet and GATA-3, resp.) were both significantly elevated in the colons of TRAF5 KO mice treated with DSS (Figure 3). Additionally, our data also showed an enhancement of IL-17a transcription in DSS-induced TRAF5-deficient animals (Figure 3). However, unexpectedly, we found no significant difference in the mRNA levels of Th17-associated transcription factors, including ROR-*α* and ROR-*γ*t, between DSS-fed KO and WT mice (Figure 3). As a result, we hypothesized that the elevated IL-17a in the colons of DSS-induced TRAF5-deficient mice might be secreted by a type of cells other than Th17 cells. Nonetheless, no difference was found in the mRNA levels of any of the cytokines or transcription factors between untreated KO and WT mice.

**Figure 4:**
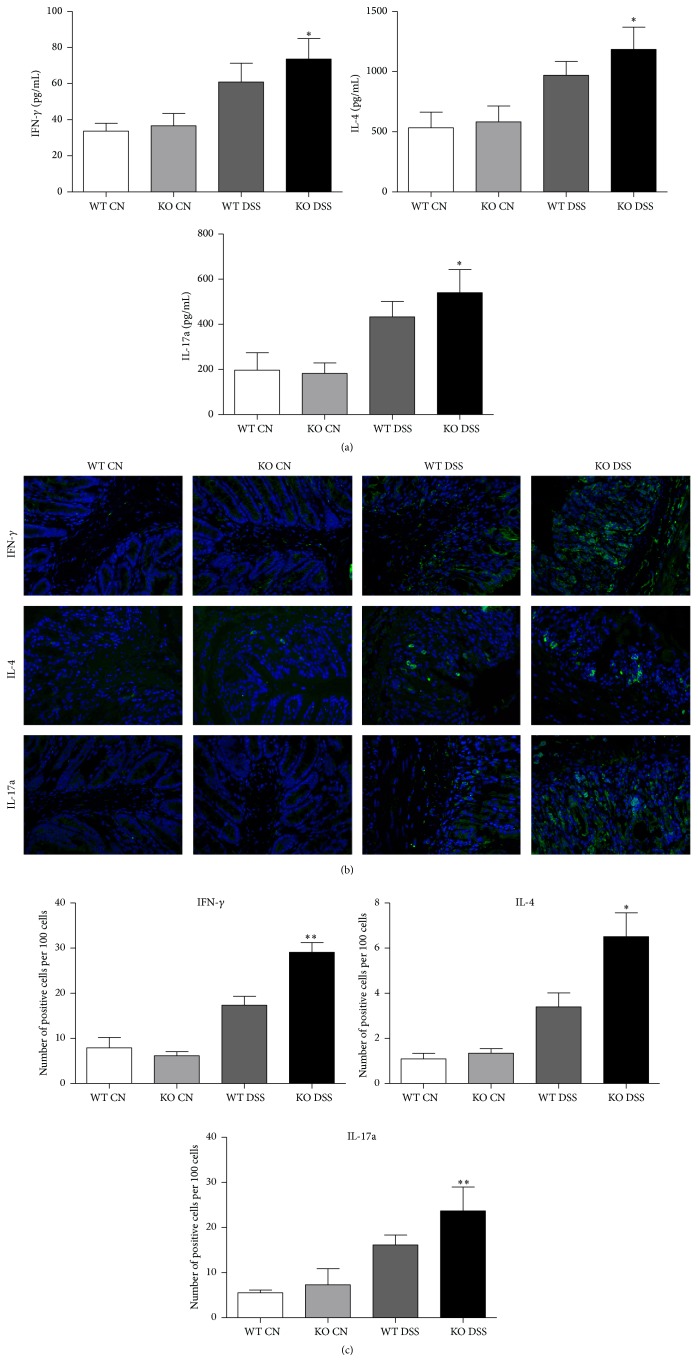
Deletion of TRAF5 increases the protein expressions of the cytokines IFN-*γ*, IL-4, and IL-17a in the colons of mice induced by DSS. (a) The production of IFN-*γ*, IL-4, and IL-17a in the colons of DSS-induced mice or control mice was examined by ELISA (*N* = 6 per group). (b) Representative immunofluorescence staining for IFN-*γ*, IL-4, and IL-17a in the colons of TRAF5 KO and WT mice (magnification 400x). (c) Quantification of positive cells (*N* = 4 per group). The data are representative of 3 independent experiments (mean ± SD). ^*∗*^ *p* < 0.05 versus the DSS-treated WT group; ^*∗∗*^ *p* < 0.01 versus the DSS-treated WT group.

Moreover, the protein levels of the cytokines IFN-*γ*, IL-4, and IL-17a were detected by ELISA and immunofluorescence staining. Our data indicated that TRAF5 deficiency could significantly enhance the protein levels of IFN-*γ*, IL-4, and IL-17a induced by DSS (Figure 4), which was in accordance with the qRT-PCR results mentioned above.

### 3.3. TRAF5 Deficiency Increases the Frequencies of Th2 and IFN-*γ*/IL-17a-Coproducing CD4^+^ T Cells in the Lamina Propria of DSS-Induced Mice

To further examine whether TRAF5 deficiency could affect the composition of CD4^+^ Th cell subsets, the surface expression of the CD4 molecule and the intracellular expression of IL-4, IFN-*γ*, and IL-17a were examined in LPMCs isolated from the colons of mice treated with DSS.

**Figure 5:**
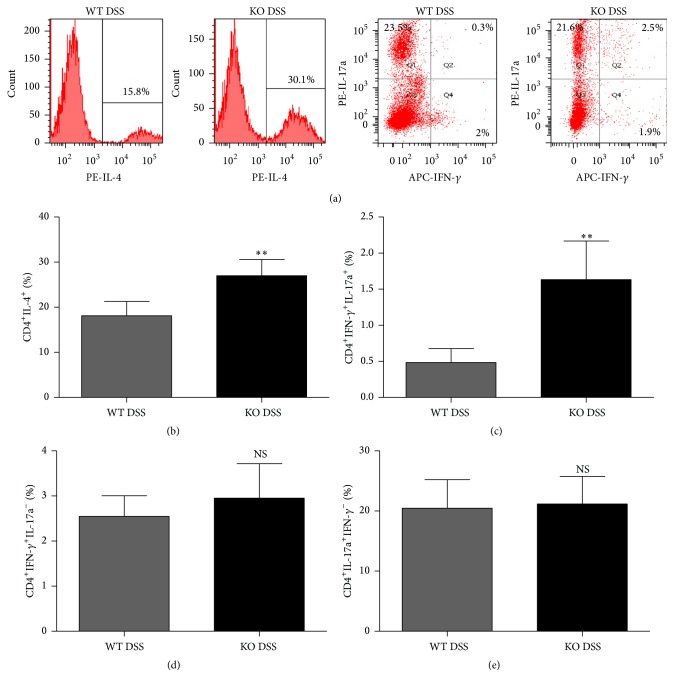
Increased proportions of Th2 and IFN-*γ*/IL-17a-coproducing CD4^+^ T cells in the colons of TRAF5-deficient animals induced by DSS. LPMCs obtained from the colons of DSS-fed KO and WT mice were stimulated with PMA/ionomycin and subjected to surface staining for CD4. After fixation and permeabilization, intracellular staining for IL-4, IFN-*γ*, and IL-17a was performed as described in Section 2. CD4^+^ T cells were then gated and analyzed. (a) Representative flow plots. The percentages of (b) Th2 cells (CD4^+^IL-4^+^), (c) IFN-*γ*/IL-17a-coproducing CD4^+^ T cells (CD4^+^IFN-*γ* ^+^IL-17a^+^), (d) classical Th1 cells (CD4^+^IFN-*γ* ^+^IL-17a^−^), and (e) classical Th17 cells (CD4^+^IL-17a^+^IFN-*γ* ^−^) are presented as the mean ± SD. The data are representative of 3 independent experiments. *N* = 6 per group. ^*∗∗*^ *p* < 0.01 versus the DSS-treated WT group. NS: not significant versus the DSS-treated WT group.

As shown in Figures 5(a) and 5(b), the percentage of CD4^+^IL-4^+^ Th2 cells was significantly increased in TRAF5-deficient mice. Meanwhile, TRAF5 deficiency also enhanced the frequency of IFN-*γ*/IL-17a-coproducing CD4^+^ T cells (Figures 5(a) and 5(c)). However, there was no significant difference in the percentages of classical Th1 and Th17 cells, characterized as CD4^+^IFN-*γ* ^+^IL-17a^−^ and CD4^+^IL-17a^+^IFN-*γ* ^−^, respectively, between KO and WT mice after DSS administration (Figures 5(a), 5(d), and 5(e)). Taken together, these results indicated that TRAF5 deficiency could markedly promote the generation of Th2 and IFN-*γ*/IL-17a-coproducing CD4^+^ T cells in response to DSS and that the elevated levels of the cytokine IL-17a, and even IFN-*γ*, in the colons of DSS-fed KO mice might be secreted by those IFN-*γ* ^+^IL-17a^+^ double-positive cells.

### 3.4. Deletion of TRAF5 Enhances the NF-*κ*B Signaling Pathway in Inflamed Colons

The colons of TRAF5 KO and WT mice were isolated for Western blot analysis to explore the potential molecular mechanisms underlying TRAF5 deficiency-mediated exacerbation of DSS-induced colitis. Because NF-*κ*B is one of the major components mediating IBDs, it plays important roles in the functional divergence and development of Th cell subsets [26, 27]. Thus, we examined the phosphorylated and total protein levels of p65 and I*κ*B*α* in the colons of KO and WT mice.

**Figure 6:**
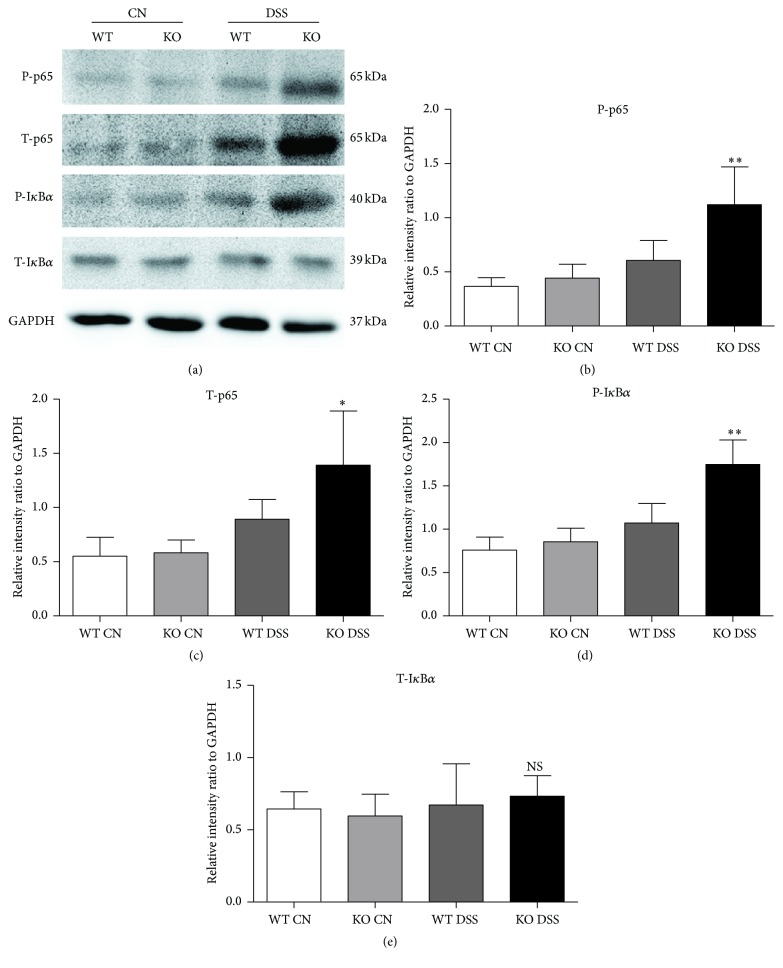
Deficiency in TRAF5 augments the activation of NF-*κ*B signaling. The phosphorylated and total protein expression levels of p65 and I*κ*B*α* were detected in the colons of TRAF5 KO and WT mice. (a) Representative blots. ((b), (c), (d), and (e)) Quantitative results. The data are representative of 3 independent experiments (mean ± SD). *N* = 4 per group. ^*∗*^ *p* < 0.05 versus the DSS-treated WT group; ^*∗∗*^ *p* < 0.01 versus the DSS-treated WT group. NS: not significant versus the DSS-treated WT group.

**Figure 7:**
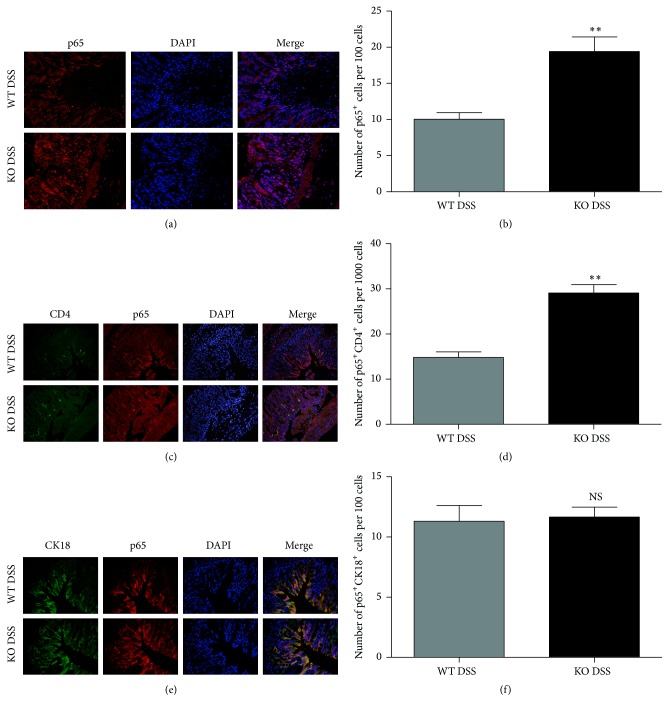
TRAF5 deficiency significantly enhances the activation of p65 in CD4^+^ T cells after the administration of DSS. (a) Representative immunofluorescence staining for p65. The p65-labeled cells show red fluorescence, and the nuclei show blue fluorescence (magnification 400x). (b) Quantification of p65 positive cells. (c) Representative immunofluorescence staining for CD4 and p65. The CD4-labeled cells show green fluorescence, p65-labeled cells show red fluorescence, and the nuclei show blue fluorescence (magnification 400x). (d) Quantification of p65^+^CD4^+^ cells. (e) Representative immunofluorescence staining for CK18 and p65. The CK18-labeled cells show green fluorescence, p65-labeled cells show red fluorescence, and the nuclei show blue fluorescence (magnification 400x). (f) Quantification of p65^+^CK18^+^ cells. The data are representative of 3 independent experiments (mean ± SD). *N* = 4 per group. ^*∗∗*^ *p* < 0.01 versus the DSS-treated WT group. NS: not significant versus the DSS-treated WT group.

Consistent with the alteration of total p65, the level of phosphorylated p65 was significantly increased in the colons of TRAF5 KO mice compared with WT mice after DSS induction (Figures 6(a), 6(b), and 6(c)). As another important mediator of the NF-*κ*B pathway, phosphorylated I*κ*B*α* was also markedly enhanced in the colons of TRAF5-deficient animals in response to DSS (Figures 6(a) and 6(d)). Nevertheless, there was no difference in the baseline activity of NF-*κ*B signaling between untreated KO and WT mice. Similarly, immunofluorescence staining of colon sections showed that TRAF5 deficiency markedly enhanced the nuclear localization of p65 compared with that in DSS-induced WT mice (Figures 7(a) and 7(b)). Therefore, these results demonstrated that TRAF5 deficiency could significantly promote the activation of NF-*κ*B signaling after DSS administration.

In addition to immune cells' function, the activation of NF-*κ*B is known to play an essential role in epithelial restitution from injury or inflammation. To clarify if the activation of NF-*κ*B mainly occurred in immune cells, epithelial cells, or both, colocalization of nuclear p65 with immune or epithelial cell makers (CD4 and CK18, resp.) was also performed in our study. As shown in Figures 7(c) and 7(d), the percentage of p65 and CD4 double-positive cells was higher in DSS-induced KO mice in comparison with WT mice. However, there was no significant difference in the rate of p65^+^CK18^+^ cells between the two groups (Figures 7(e) and 7(f)). Taken together, these results indicated that the elevated activation of NF-*κ*B in the colons of TRAF5 KO mice mainly occurred in immune cells but not the epithelial cells.

## 4. Discussion

In our experiments, we obtained the novel finding that TRAF5 participates in the regulation of local intestinal inflammation. The loss-of-function experiments showed that TRAF5 deficiency could exacerbate DSS-induced colitis, characterized by striking body weight loss, a significantly increased DAI score, dramatic shortening of the colon, and more severe histological injury. Furthermore, we examined the activities of MPO, an enzyme found mainly in granulocytes, and found that TRAF5 KO mice exhibited higher levels of MPO activities than WT mice did after DSS induction. Our data therefore demonstrated that the deletion of TRAF5 could significantly aggravate the severity of DSS-induced colitis.

Because the adaptor TRAF5 has been reported to be associated with the regulation of CD4^+^ Th cell immunity [15, 21, 22], we next examined the levels of typical cytokines and transcription factors of Th cell subsets (Th1, Th2, and Th17) in the colons of KO and WT mice. Our results indicated that the expression levels of the cytokines IFN-*γ*, IL-4, and IL-17a were all significantly elevated in TRAF5-deficient mice in comparison with WT mice induced by DSS. Additionally, our data suggested that TRAF5 deficiency could significantly enhance the mRNA levels of the transcription factors T-bet and GATA-3. However, to our surprise, we found no significant difference in the levels of any specific Th17 transcription factors between the two groups of mice. Hence, we hypothesized that the elevated IL-17a in the colons of DSS-fed KO mice might be secreted by a type of cells other than Th17 cells. Apart from Th17 cells, one unique type of cells, namely, IFN-*γ*/IL-17a-coproducing CD4^+^ T cells, can also secrete IL-17a [28–31], and these distinct populations piqued our interest.

To our knowledge, IFN-*γ*/IL-17a-coproducing CD4^+^ T cells represent a group of cells that are transformed from Th1 or Th17 cells. Although classical Th1 and Th17 cells are mainly IFN-*γ* and IL-17a single-positive, respectively, this group of cells is IFN-*γ* and IL-17a double-positive [28–31]. Increasing evidence has demonstrated that these populations play essential roles in the pathogenesis of IBDs and other autoimmune diseases [29, 32, 33]. To confirm our hypothesis and further clarify the mechanism, the percentages of CD4^+^ Th cell subpopulations were analyzed by flow cytometry.

Our data suggested that the percentage of IFN-*γ*/IL-17a-coproducing CD4^+^ T cells was significantly elevated in the LPMCs of TRAF5-deficient mice compared with those of WT mice following DSS induction. However, the frequencies of classical Th1 and Th17 cells were not significantly different between the two groups. Based on these findings, we assumed that the increase in IFN-*γ* ^+^IL-17a^+^ double-positive cells might play a critical role in the enhanced production of IL-17a and IFN-*γ* in the colons of TRAF5-deficient animals in response to DSS. Although the plasticity of Th17 cells is widely accepted, the flexibility of Th1 cells is less understood. Liu and his colleagues found that Th1 cells could transform into IFN-*γ*/IL-17a-coproducing CD4^+^ T cells or even Th17 cells and that this transformation was dependent on the expression of the transcription factor T-bet [30]. Similarly, our experiments revealed an increased percentage of IFN-*γ*/IL-17a-coproducing CD4^+^ T cells and elevated expression of T-bet in DSS-induced KO mice. Therefore, we propose that the increased percentage of IFN-*γ* ^+^IL-17a^+^ double-positive cells in the colons of DSS-fed KO mice was probably due to transformation of Th1 cells. However, the precise mechanism is unclear and will require further investigation.

Furthermore, our data showed an elevated frequency of Th2 (CD4^+^ IL-4^+^) cells in the colons of TRAF5-deficient mice after DSS induction, which was consistent with the alteration of cytokine and transcription factor profiles. Several studies have suggested that overexpression of GATA-3 in T cells could markedly aggravate the severity of DSS-induced colitis in mice and that IL-4-deficient mice develop less severe DSS-induced colitis than WT mice do [34, 35]. Thus, we had reason to believe that the elevated level of Th2 cells and their specific cytokine IL-4 in the colons of TRAF5 KO mice might play proinflammatory roles during the development of DSS-induced colitis.

The NF-*κ*B family contains five different members, including cRel, RelA (p65), RelB, NF-*κ*B1 (p50), and NF-*κ*B2 (p52) [36]. Under resting conditions, NF-*κ*B is mainly inactivated and kept in the cytoplasm by interaction with the inhibitory protein I*κ*B. Upon stimulation, a series of kinases activates and phosphorylates I*κ*B, leading to its rapid degradation by ubiquitination. Finally, the released NF-*κ*B passes into the nucleus, where it binds to the specific sequences of target genes [37, 38]. In our study, we observed that TRAF5 deficiency increased the phosphorylation of p65 and I*κ*B*α* in response to DSS induction. In accordance with the Western blot data, the immunofluorescence staining also showed enhancement of the nuclear localization of p65 in DSS-fed KO mice. These results suggested that the deletion of TRAF5 could significantly promote the activation and translocation of NF-*κ*B in the murine colitis model. Although our data have demonstrated that NF-*κ*B is activated in TRAF5 knockout mice during DSS colitis, it is not clear if this activation occurs in immune cells, epithelial cells, or both. In order to figure out this puzzle, colocalization of nuclear p65 with immune or epithelial cell makers was also performed in our study. And the results suggested that the elevated activation of NF-*κ*B in the colons of TRAF5 KO mice mainly occurred in immune cells but not the epithelial cells. Notably, NF-*κ*B signaling has been shown to mediate numerous aspects of Th cell development and differentiation [26, 39, 40]. We therefore assumed that the influence of TRAF5 deficiency on the responses of Th cell subsets in our experiments might be NF-*κ*B-dependent. To our knowledge, previous studies have demonstrated that the adaptor TRAF5 participates in the activation of NF-*κ*B signaling [18, 19, 41]. Our study challenged this common view by suggesting that NF-*κ*B was activated in the colons of TRAF5-deficient mice induced by DSS. In agreement with our findings, Bian et al. also found enhancement of NF-*κ*B in the hearts of TRAF5-deficient animals under pressure overload [42]. Thus, we propose that TRAF5's effect on NF-*κ*B signaling might be tissue and disease specific.

In summary, our study identified TRAF5 as an anti-inflammatory modulator in murine experimental colitis. Under intestinal inflammatory conditions, TRAF5 deficiency enhances the responses of Th2 and IFN-*γ*/IL-17a-coproducing CD4^+^ T cells. Meanwhile, the activation of NF-*κ*B in CD4^+^ T cells is also enhanced. These results suggest that TRAF5 deficiency significantly aggravates DSS-induced colitis, most likely by regulating Th cell-mediated inflammation. Targeting of TRAF5 may thus provide a promising novel strategy for the treatment of human IBDs.

## Figures and Tables

**Table 1:** Disease activity index (DAI) scoring criteria.

Score	Body weight loss (%)	Stool consistency	Rectal bleeding
0	None	Normal	Normal
1	1–5	N/A	N/A
2	5–10	Loose	Occult bleeding
3	10–20	N/A	N/A
4	>20	Diarrhea	Gross bleeding

**Table 2:** Histological scoring system used in this study.

Point	Epithelial damage	Inflammatory cell infiltration
0	Normal morphology	No infiltrate
1	Loss of goblet cells	Infiltrate around crypt base
2	Loss of goblet cells in large areas	Infiltrate reaching the L. muscularis mucosae
3	Loss of crypts	Extensive infiltration reaching the L. muscularis mucosae and thickening of the mucosa with abundant edema
4	Loss of crypts in large areas	Infiltration of the L. submucosa

**Table 3:** The gene-specific primer pairs list in the study.

Primer names	Primer sequences (5′-3′)
TNF-*α*-F	CCACGTCGTAGCAAACCACCAA
TNF-*α*-R	TTTGAGATCCATGCCGTTGGCCA
IFN-*γ*-F	CGCTACACACTGCATCTTGG
IFN-*γ*-R	GCTTTCAATGACTGTGCCGT
IL-4-F	CGAAGAACACCACAGAGAGTGAGC
IL-4-R	GACTCATTCATGGTGCAGCTTATCG
IL-17a-F	TCCACCGCAATGAAGACCCTGA
IL-17a-R	TCCAGCTTTCCCTCCGCATTGA
IL-22-F	GTGCTCAACTTCACCCTGGA
IL-22-R	AGGAGCTGAGCTGATTGCTG
T-bet-F	TGTTCCCAGCCGTTTCTACC
T-bet-R	GCTCGGAACTCCGCTTCATA
GATA-3-F	GAAGGCAGGGAGTGTGTGAA
GATA-3-R	TCGCTTGGGCTTGATAAGGG
ROR-*α*-F	GAGCTCCAGCAGATAACGTG
ROR-*α*-R	GCAAACTCCACCACATACTGG
ROR-*γ*t-F	GCTGCGACTGGAGGACCTTCTA
ROR-*γ*t-R	CGCTCCCACATCTCCCACATTG
GAPDH-F	ACTCCACTCACGGCAAATTC
GAPDH-R	TCTCCATGGTGGTGAAGACA

## List of Abbreviations

IBDs: Inflammatory bowel diseases
CD: Crohn's disease
UC: Ulcerative colitis
Th: T helper
DSS: Dextran sulfate sodium
TRAF5: Tumor necrosis factor receptor-associated factor 5
TLR: Toll-like receptor
DAI: Disease activity index
DAPI: 4′,6-Diamidino-2-phenylindole
LPMC: Lamina propria mononuclear cell.

## References

[1] Kaser A., Zeissig S., Blumberg R. S. (2010). Inflammatory bowel disease. *Annual Review of Immunology*.

[2] Strober W., Fuss I. J. (2011). Proinflammatory cytokines in the pathogenesis of inflammatory bowel diseases. *Gastroenterology*.

[3] Maynard C. L., Weaver C. T. (2009). Intestinal effector T cells in health and disease. *Immunity*.

[4] Fujino S., Andoh A., Bamba S. (2003). Increased expression of interleukin 17 in inflammatory bowel disease. *Gut*.

[5] Neurath M. F., Finotto S., Glimcher L. H. (2002). The role of TH1/TH2 polarization in mucosal immunity. *Nature Medicine*.

[6] Fuss I. J., Neurath M., Boirivant M. (1996). Disparate CD4+ Lamina Propria (LP) lymphokine secretion profiles in inflammatory Bowel disease: Crohn's disease LP cells manifest increased secretion of IFN-*γ*, whereas ulcerative colitis LP cells manifest increased secretion of IL-5. *Journal of Immunology*.

[7] Wirtz S., Neufert C., Weigmann B., Neurath M. F. (2007). Chemically induced mouse models of intestinal inflammation. *Nature Protocols*.

[8] Oh S. Y., Cho K.-A., Kang J. L., Kim K. H., Woo S.-Y. (2014). Comparison of experimental mouse models of inflammatory bowel disease. *International Journal of Molecular Medicine*.

[9] Bradley J. R., Pober J. S. (2001). Tumor necrosis factor receptor-associated factors (TRAFs). *Oncogene*.

[10] Xie P., Poovassery J., Stunz L. L. (2011). Enhanced Toll-like receptor (TLR) responses of TNFR-associated factor 3 (TRAF3)-deficient B lymphocytes. *Journal of Leukocyte Biology*.

[11] Clark K., Takeuchi O., Akira S., Cohen P. (2011). The TRAF-associated protein TANK facilitates cross-talk within the I*κ*B kinase family during Toll-like receptor signaling. *Proceedings of the National Academy of Sciences of the United States of America*.

[12] Zhou Q., Geahlen R. L. (2009). The protein-tyrosine kinase Syk interacts with TRAF-interacting protein TRIP in breast epithelial cells. *Oncogene*.

[13] Au P.-Y. B., Yeh W.-C. (2007). Physiological roles and mechanisms of signaling by TRAF2 and TRAF5. *Advances in Experimental Medicine and Biology*.

[14] Zotti T., Vito P., Stilo R. (2012). The seventh ring: exploring TRAF7 functions. *Journal of Cellular Physiology*.

[15] Hildebrand J. M., Yi Z., Buchta C. M., Poovassery J., Stunz L. L., Bishop G. A. (2011). Roles of tumor necrosis factor receptor associated factor 3 (TRAF3) and TRAF5 in immune cell functions. *Immunological Reviews*.

[16] Tada K., Okazaki T., Sakon S. (2001). Critical roles of TRAF2 and TRAF5 in tumor necrosis factor-induced NF-*κ*B activation and protection from cell death. *The Journal of Biological Chemistry*.

[17] Nakano H., Sakon S., Koseki H. (1999). Targeted disruption of Traf5 gene causes defects in CD40- and CD27-mediated lymphocyte activation. *Proceedings of the National Academy of Sciences of the United States of America*.

[18] Akiba H., Nakano H., Nishinaka S. (1998). CD27, a member of the tumor necrosis factor receptor superfamily, activates NF-*κ*B and stress-activated protein kinase/c-Jun N-terminal kinase via TRAF2, TRAF5, and NF-*κ*B-inducing kinase. *Journal of Biological Chemistry*.

[19] Nakano H., Oshima H., Chung W. (1996). TRAF5, an activator of NF-*κ*B and putative signal transducer for the lymphotoxin-*β* receptor. *The Journal of Biological Chemistry*.

[20] Buchta C. M., Bishop G. A. (2014). TRAF5 negatively regulates TLR signaling in B lymphocytes. *Journal of Immunology*.

[21] So T., Salek-Ardakani S., Nakano H., Ware C. F., Croft M. (2004). TNF receptor-associated factor 5 limits the induction of Th2 immune responses. *Journal of Immunology*.

[22] Kraus Z. J., Haring J. S., Bishop G. A. (2008). TNF receptor-associated factor 5 is required for optimal T cell expansion and survival in response to infection. *Journal of Immunology*.

[23] Cooper H. S., Murthy S. N. S., Shah R. S., Sedergran D. J. (1993). Clinicopathologic study of dextran sulfate sodium experimental murine colitis. *Laboratory Investigation*.

[24] Obermeier F., Kojouharoff G., Hans W., Schölmerich J., Gross V., Falk W. (1999). Interferon-gamma (IFN-*γ*)- and tumour necrosis factor (TNF)-induced nitric oxide as toxic effector molecule in chronic dextran sulphate sodium (DSS)-induced colitis in mice. *Clinical and Experimental Immunology*.

[25] Weigmann B., Tubbe I., Seidel D., Nicolaev A., Becker C., Neurath M. F. (2007). Isolation and subsequent analysis of murine lamina propria mononuclear cells from colonic tissue. *Nature Protocols*.

[26] Oh H., Ghosh S. (2013). NF-*κ*B: roles and regulation in different CD4+ T-cell subsets. *Immunological Reviews*.

[27] Atreya I., Atreya R., Neurath M. F. (2008). NF-*κ*B in inflammatory bowel disease. *Journal of Internal Medicine*.

[28] Harbour S. N., Maynard C. L., Zindl C. L., Schoeb T. R., Weaver C. T. (2015). Th17 cells give rise to Th1 cells that are required for the pathogenesis of colitis. *Proceedings of the National Academy of Sciences of the United States of America*.

[29] Globig A.-M., Hennecke N., Martin B. (2014). Comprehensive intestinal T helper cell profiling reveals specific accumulation of IFN-*γ*+IL-17+coproducing CD4+ T cells in active inflammatory bowel disease. *Inflammatory Bowel Diseases*.

[30] Liu H.-P., Cao A. T., Feng T. (2015). TGF-*β* converts Th1 cells into Th17 cells through stimulation of Runx1 expression. *European Journal of Immunology*.

[31] Wang Y., Godec J., Ben-Aissa K. (2014). The transcription factors T-bet and Runx are required for the ontogeny of pathogenic interferon-*γ*-producing T helper 17 cells. *Immunity*.

[32] Ghoreschi K., Laurence A., Yang X.-P. (2010). Generation of pathogenic TH 17 cells in the absence of TGF-*β* 2 signalling. *Nature*.

[33] Martin-Orozco N., Chung Y., Chang S. H., Wang Y.-H., Dong C. (2009). Th17 cells promote pancreatic inflammation but only induce diabetes efficiently in lymphopenic hosts after conversion into Th1 cells. *European Journal of Immunology*.

[34] Stevceva L., Pavli P., Husband A., Ramsay A., Doe W. F. (2001). Dextran sulphate sodium-induced colitis is ameliorated in interleukin 4 deficient mice. *Genes and Immunity*.

[35] Okamura M., Yoh K., Ojima M., Morito N., Takahashi S. (2014). Overexpression of GATA-3 in T cells accelerates dextran sulfate sodium-induced colitis. *Experimental Animals*.

[36] Barnes P. J., Karin M. (1997). Nuclear factor-*κ*B—a pivotal transcription factor in chronic inflammatory diseases. *The New England Journal of Medicine*.

[37] Bonizzi G., Karin M. (2004). The two NF-*κ*B activation pathways and their role in innate and adaptive immunity. *Trends in Immunology*.

[38] Wei J., Feng J. (2010). Signaling pathways associated with inflammatory bowel disease. *Recent Patents on Inflammation and Allergy Drug Discovery*.

[39] Das J., Chen C.-H., Yang L., Cohn L., Ray P., Ray A. (2001). A critical role for NF-*κ*B in Gata3 expression and TH2 differentiation in allergic airway inflammation. *Nature Immunology*.

[40] Gerondakis S., Siebenlist U. (2010). Roles of the NF-kappaB pathway in lymphocyte development and function. *Cold Spring Harbor Perspectives in Biology*.

[41] Wang L., Lu Y., Guan H. (2013). Tumor necrosis factor receptor-associated factor 5 is an essential mediator of ischemic brain infarction. *Journal of Neurochemistry*.

[42] Bian Z., Dai J., Hiroyasu N. (2014). Disruption of tumor necrosis factor receptor associated factor 5 exacerbates pressure overload cardiac hypertrophy and fibrosis. *Journal of Cellular Biochemistry*.

